# A Neuro-Inspired Rate-Encoded Descriptor for High-Speed Asynchronous Robotic Vision †

**DOI:** 10.3390/s26165311

**Published:** 2026-08-21

**Authors:** Shane Harrigan, Sonya Coleman, Dermot Kerr, Pratheepan Yogarajah, Chengdong Wu, Zheng Fang

**Affiliations:** 1 Intelligent Systems Research Centre, Ulster University, Londonderry BT48 7JL, UK; 2 Faculty of Robot Science and Engineering, Northeastern University, Shenyang 110819, China

**Keywords:** asynchronous processing, bio-inspired algorithms, event-based sensors (DVS), feature extraction, motion classification, neural coding, neuromorphic computing, pattern recognition, rate encoding, robotic vision, RoShamBo

## Abstract

**Highlights:**

**What are the main findings?**

- Introduced P-TED, a pure event descriptor for asynchronous neuromorphic data.
- Achieved 2.7 ms latency, 3.8× faster than state-of-the-art methods on DVS datasets.

**What are the implications of the main findings?**

- Resolves directional disambiguation against spatial mirroring in robotic perception.
- Enables high-speed, real-time recognition of non-rigid gestures for robotic hands.

**Abstract:**

This paper presents the Post-Stimulus Time-Dependent Event Descriptor (P-TED), a novel “pure event” feature descriptor designed for neuromorphic vision data. Unlike conventional frame-based approaches or hybrid methods that transform event data into intermediate representations, P-TED operates directly on asynchronous event streams, thereby preserving the intrinsic low-latency and high-temporal-resolution advantages of event-based sensors. The descriptor integrates two complementary feature sets: a motion feature vector, which aggregates spatial relationships within a Moore neighbourhood to quantify stimulus direction, and a pattern feature vector, which employs rate encoding to capture temporal excitation signatures. The efficacy of the P-TED framework is validated through three distinct experiments: object and character recognition (MNIST-DVS and CIFAR10-DVS), mobile robot movement analysis, and complex non-rigid robotic hand gesture recognition (RoShamBo). Experimental results demonstrate that the P-TED achieves a significant reduction in classification latency, requiring only 2.7 ms compared to the 10.3 ms recorded by the state-of-the-art Distribution-Aware Retinal Transform (DART) framework. Additionally, P-TED exhibits superior robustness in disambiguating symmetric and mirrored motions, as well as in maintaining stability under non-linear fluctuations in event density caused by changing scale. This work establishes P-TED as a high-speed, computationally efficient, and explainable solution for real-time neuromorphic robotic vision systems.

## 1. Introduction

In the early 1990s, the first artificial retina, often termed the “silicon retina” was designed [1]. This pioneering device utilised analogue electrical circuits to mimic the biological functions of photoreceptive and non-photoreceptive cells within the retina. While event-based vision sensors remained primarily experimental for decades, the emergence of commercial-grade hardware, such as the Dynamic Vision Sensor (DVS) [2], has established the burgeoning sub-field of neuromorphic or event-based computer vision.

Unlike conventional “frame-based” imaging devices that capture data at fixed synchronous intervals, the DVS is modelled on the asynchronous nature of biological photosensitive cells. These sensors emit a discrete signal, or “event”, only when a change in log-luminance exceeds a predefined threshold. Devices incorporating DVS technology, such as the DAVIS240C [3] and the DVS128 [2], produce an asynchronous stream of data comparable to neural spike trains. A significant advantage of this approach is the inclusion of a polarity value for each event, which allows the sensor to distinguish between an increase and a decrease in luminance with high temporal resolution and low power consumption.

Initial research into processing event-based vision data focused on feature extraction, particularly the detection of corners within event streams [4]. Common techniques rely upon machine learning [5,6,7], clustering [8,9,10], or the conversion of event data into frame-based representations and contrast maps to apply established image processing algorithms such as the Harris [11] or FAST [12] algorithms. While frame-based vision devices are restricted to a synchronised rate of data emission (e.g., 30 frames per second), event-based sensors emit data at microsecond resolution. These sensors allow for the extraction of reliable low-level features without suffering from the aperture problem [13]. Conversely, the latency inherent in synchronised data emission results in information loss due to temporal sampling limitations, which can be problematic for high-speed robotics [14]. Consequently, contemporary research is increasingly exploring machine learning architectures tailored for event data, including Support Vector Machines (SVMs) [15,16], Random Forests [17], Convolutional Neural Networks (CNNs) [18,19], Spiking Neural Networks (SNN) [20,21,22], and Recurrent Neural Networks (RNN) [23,24].

The inherent trade-off between processing speed and accuracy in conventional computer vision persists in the event-based domain. When employing heavy computational methods such as machine learning, the intrinsic benefits of event-based systems, namely, low power consumption and minimal latency, are often diminished. Consequently, it is preferable to process event data using alternative paradigms that preserve these architectural advantages. Neuroscience research offers several models for how action potentials, and, by extension, events, encode information. For instance, the latency of a neuron’s response to a stimulus, termed ‘time-to-first-spike’ encoding [25], has been shown to contain sufficient information for a coarse reconstruction of the visual stimulus [26]. Other schemes focus on the correlation between multiple spike trains, which is fundamental to neuronal communication [27]. Incorporating these neuroscientific principles is therefore critical when developing efficient encoding techniques for event-based motion data.

This paper introduces an event-based feature descriptor that operates directly on raw event streams without conversion into conventional frames. The proposed method, termed the ‘Post-Stimulus Time-Dependent Event Descriptor’ (P-TED), extracts two distinct feature types from asynchronous data. P-TED is inspired by neuroscientific research concerning the encoding of time-dependent neural activity into feature vector representations [28]. In this work, P-TED is integrated with a complementary event-based motion classification framework [29]. Its performance is evaluated against state-of-the-art event-based descriptors as well as established frame-based descriptors for object detection tasks.

The primary contributions to the field of neuromorphic vision are four-fold:

- Introduction of P-TED: A novel, pure event descriptor formed directly from event data streams without remapping them into 2D frames, ensuring minimal computational overhead and preservation of temporal sparsity.
- Dual-Feature Representation: A unique combination of a motion feature vector (*V*), which measures stimulus direction across cardinalities using a Moore neighbourhood (the eight surrounding pixels), and a pattern feature vector (*G*), which captures event pixel excitation patterns via rate encoding.
- Novel Classification Framework: A high-speed framework utilising a temporal sliding window to compare Motions of Interest (MoI) against live event streams in real time, facilitating high-speed robotic interaction.
- Empirical Validation: Demonstrations of accuracy and speed over established frameworks across three domains: object recognition (MNIST-DVS), mobile robot motion (Summit XL), and complex robotic hand symbols (RoShamBo).

P-TED builds upon earlier conceptual work in space–time templates [29] by formalising the mathematical correlation of events and establishing a benchmarking foundation for evaluating advanced neural coding strategies. Specifically, this work demonstrates the efficacy of manual feature engineering over deep learning in scenarios constrained by strict latency and power requirements.

## 2. Materials and Methods

### 2.1. Post-Stimulus Time-Dependent Event Descriptor

The proposed Post-Stimulus Time-Dependent Event Descriptor (P-TED) is a purely event-based descriptor designed to capture two distinct feature types from asynchronous data streams. The first component, the motion feature vector (*V*), measures the direction of stimulus movement, while the second, the pattern feature vector (*G*), captures the temporal excitation patterns and overall activation region of the stimulus. These vectors are combined to form the final P-TED representation. Typically, event-based vision devices communicate via the Address-Event Representation (AER) protocol [30]. In this framework, an event stream *S* consists of discrete events $e_{i}$, where each event is defined as a tuple $e_{i}=\langle t_{i},\langle x_{i},y_{i}\rangle ,p_{i}\rangle$. Here, *i* represents the event index, *t* denotes the detection timestamp, 〈x,y〉 indicates the spatial coordinates of the pixel sensing a change in luminance, and p∈{1,−1} represents the polarity, indicating an increase or decrease in light intensity. Three sequential steps are required to construct the P-TED from event data streams: event correlation, motion feature vector formation, and pattern feature vector formation.

Event-based sensors generate an asynchronous stream *S*, defined as an ordered sequence of *N* events, $S={\left\{e_{i}\right\}}_{i=1}^{N}$. Each individual event $e_{i}$ is characterised by a tuple $\langle t_{i},l_{i},p_{i}\rangle$, where i∈{1,…,N} is the chronological index, $t_{i}$ is the timestamp of occurrence, $l_{i}=\langle x_{i},y_{i}\rangle$ represents the spatial coordinates on the pixel grid, and $p_{i}\in \left\{-1,+1\right\}$ denotes the polarity of the luminance change. To enhance technical robustness against noise, P-TED operates exclusively on a subset R⊂S of correlated events [29,31,32]. The correlation process functions as an inherent spatio-temporal noise filter. By enforcing connectivity within a Moore neighbourhood, asynchronous noise events lacking sufficient local support are excluded from the descriptor formation, thereby ensuring feature stability. Mathematically, the set of correlated pairs *R* is defined as follows:

(1) $$R=\{\left\{e_{i},e_{j}\right\}|l_{j}\in H\left(l_{i}\right),\;t_{i}-t_{j}\leq \Delta \}$$

In this formulation, $e_{i}$ represents the current event being processed at time $t_{i}$, and $e_{j}$ is a preceding target event recorded at time $t_{j}$. The term $H(l_{i})$ denotes the Moore neighbourhood [33], the set of eight pixels surrounding the spatial location $l_{i}$, and Δ represents the temporal threshold (e.g., 5 μs). In instances where multiple preceding events satisfy the spatial constraint, the neighbour with the minimum temporal distance $\delta_{t}=t_{i}-t_{j}$ is selected to form the pair. This correlation stage serves a dual purpose:

- Feature Extraction: It establishes the local motion flow required for the motion feature vector *V*.
- Asynchronous Noise Suppression: Isolated events triggered by sensor noise typically lack spatio-temporal neighbours and fail to satisfy the constraints in (1). Consequently, they are excluded from the set *R*, ensuring that P-TED is constructed only from meaningful, stimulus-driven data.

The candidate neighbours $e_{j}$ are evaluated in a clockwise sequence starting from the northern pixel $H_{N}$. If no previous event $e_{j}$ satisfies the spatio-temporal constraints, the current event $e_{i}$ is added to the time-surface T to potentially support future correlations, but it is not included in the descriptor set *R* for the current timestep.

If a target event $e_{j}$ satisfies the spatio-temporal constraints relative to the current event $e_{i}$, the pair $r_{k}=\left\{e_{i},e_{j}\right\}$ is formed and added to the set of correlated events *R*. Conversely, if no such event $e_{j}$ exists, $e_{i}$ is recorded on the time-surface T to facilitate future correlations but is excluded from *R*. The set *R* is formally expressed as follows:

(2) $$R=\{\left\{e_{i},e_{j}\right\}|\delta_{t}\leq \Delta \}$$

where $\delta_{t}=t_{i}-t_{j}$. Any event $e_{i}$ demonstrating spatial proximity without meeting the temporal threshold, or vice versa, is appended to the time-surface but omitted from the descriptor set *R*. The potential neighbours $e_{j}$ are evaluated in a clockwise sequence originating from the northern position $H_{N}$. In instances where multiple neighbours possess identical temporal distances $\delta_{t}$, the first occurrence encountered within the clockwise search is selected to form the pair in *R*.

**Figure 1:**
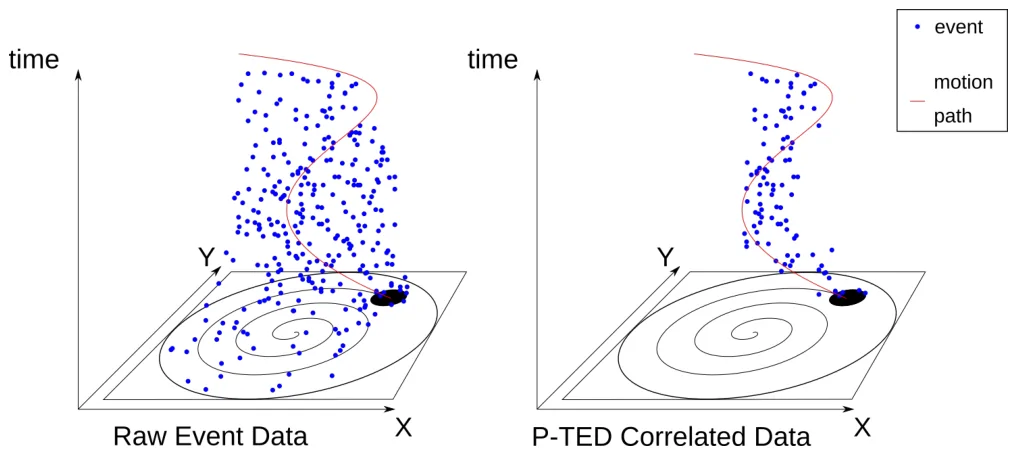
Illustration of raw asynchronous event data (**left**) compared to event data correlated and filtered within the P-TED framework (**right**).

The P-TED is applied directly to the set *R* of correlated events to extract high-level feature representations (see Figure 1). P-TED comprises two primary components: the motion direction vector (*V*) and the temporal pattern vector (*G*). While *V* characterises the directional flow of the stimulus, *G* captures the specific pattern of event pixel excitation over time.

The motion feature vector *V* quantifies the direction of stimulus movement by aggregating the spatial relationships of all correlated pairs in *R*. For each pair $\{e_{i},e_{j}\}\in R$, a directional vector is established from the current event $e_{i}$ toward its preceding neighbour $e_{j}$. We define *V* as a 1D histogram of n=8 bins, where each bin $V_{k}$ corresponds to a 45° arc representing the cardinal and intercardinal directions of the Moore neighbourhood *H*. The magnitude of each bin is calculated as the cardinality of pairs oriented toward that specific neighbour’s position:

(3) $$V=[|H_{N}|,|H_{NE}|,|H_{E}|,|H_{SE}|,|H_{S}|,|H_{SW}|,|H_{W}|,|H_{NW}|]$$

By observing these counts in a clockwise sequence starting from $H_{N}$, *V* provides an intuitive and explainable representation of the underlying visual motion. For instance, a vector V=[23,5,1,0,0,0,0,0] characterises motion that is predominantly northward with a slight bearing toward the east. This formulation ensures robustness against mirroring effects, as symmetric motions result in distinct, identifiable peaks within the *V* vector.

The pattern vector *G* captures the temporal dynamics of the event stream using a rate encoding technique [34]. Unlike frame-based methods, *G* represents the intensity of pixel excitation over time without spatial remapping, providing a time-dependent signature analogous to biological encoding schemes such as time-to-first-spike encoding [26]. For a given stream *S*, the temporal duration is partitioned into γ bins of size β. To address the ‘optimal bin size’ problem, we implement an iterative cost minimisation function based on the Poisson statistics of the correlated event pairs $r_{k}$ [35]. Let $N_{j}$ denote the number of event pairs within bin *j*. We calculate the mean *f* and variance *d* of $N_{j}$ as follows:

(4) $$f=\frac{1}{\gamma }\sum_{j=1}^{\gamma }N_{j},\;d=\frac{1}{\gamma }\sum_{j=1}^{\gamma }{\left(N_{j}-f\right)}^{2}$$

The optimal bin size β is determined by minimising the cost function C(β):

(5) $$C\left(\beta \right)=\frac{2f-d}{\beta^{2}}$$

**Figure 2:**
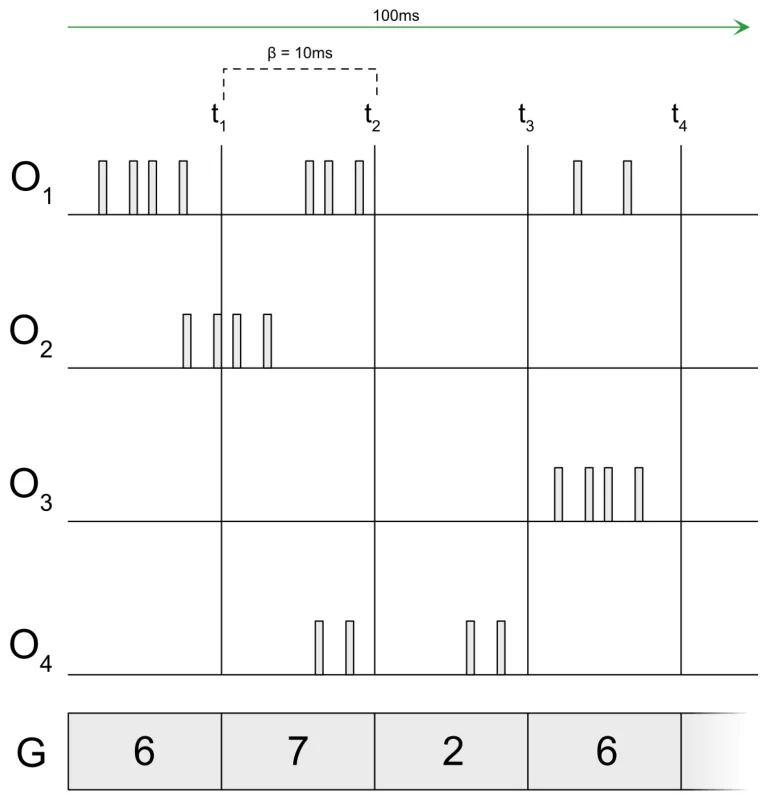
An illustration of the pattern feature vector (*G*) formation through summation with a bin size β=10 where the event pair density is aggregated to form the excitation signature.

By iteratively varying β to minimise (5), we determine the bin width that best reflects the underlying excitation activity. In practice, this formation process is performed across the entire sensor array, resulting in the aggregate descriptor $G=\{O_{0},\ldots ,O_{\phi }\}$, where ϕ=K×L represents the height and width of the event sensor array, respectively (see Figure 2). It should be noted that calculating the optimal bin size β via Equation (5) assumes stationary event statistics within the evaluation window. In environments with dynamic rate changes (e.g., rapid acceleration or varying stimulus speed), an online adaptive binning scheme can be employed, where β(t) is re-estimated over sliding temporal sub-windows based on real-time event arrival rate variance R(t).

### 2.2. P-TED Classification Framework

We present a novel classification framework designed to leverage the P-TED for object motion classification. The framework operates on a ‘Motion of Interest’ (MoI) event stream, denoted as *M*, which represents the specific motion profile to be classified. For this stream, the corresponding pattern vector M(G) and motion direction vector M(V) are computed.

**Figure 3:**
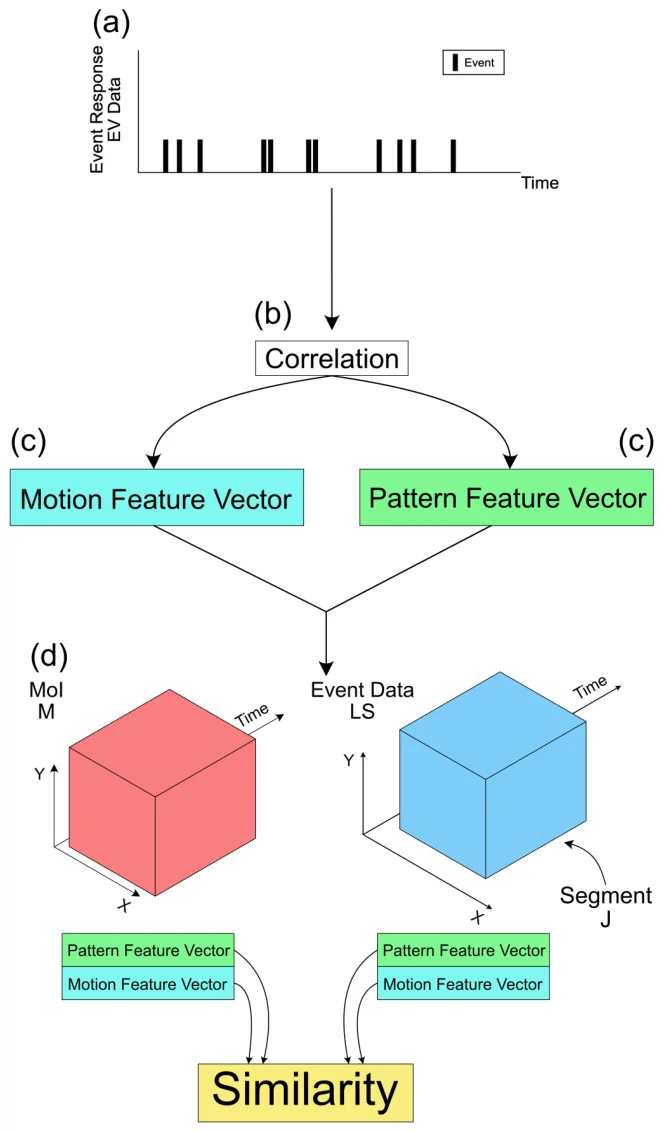
Overview of the P-TED architecture: (**a**) input event data are (**b**) correlated; (**c**) P-TED motion and pattern vectors are formulated; and (**d**) a similarity comparison is performed using Equations (6)–(8).

To perform classification on a live event stream LS, we employ a temporal sliding window to extract a segment *J*. The window size is configured such that the number of events in *J* matches the MoI size (|J|=|M|), provided the live stream is sufficiently long (|M|<|LS|). Within each window, the P-TED features J(V) and J(G) are computed and compared against the template vectors of *M*. An illustration of the overall system architecture is detailed in Figure 3.

The similarity measure Sim(V) between the motion direction feature vectors M(V) and J(V) is computed by evaluating the elementwise correspondence across the n=8 bins:

(6) $$\mathrm{Sim}\left(V\right)=\frac{1}{n}\sum_{i=1}^{n}\left\{\begin{array}{cc} 1 & \mathrm{if}\;M{\left(V\right)}_{i}=J{\left(V\right)}_{i}\\ 0 & \mathrm{if}\;M{\left(V\right)}_{i}\neq J{\left(V\right)}_{i} \end{array}\right.$$

The result is a value in the range [0,1], where 1 represents a perfect alignment of motion directions and 0 indicates no similarity between the MoI and the live segment. Equation (6) provides a lightweight discrete matching condition, where we match each element of the feature vectors in turn, and therefore is suitable for low-power, fixed-point hardware implementations. In other implementations, continuous distance metrics can also be employed for soft matching. For example, Cosine Similarity $S_{\cos}\left(M\left(V\right),J\left(V\right)\right)=\frac{M(V)\cdot J(V)}{{∥M\left(V\right)∥}_{2}{∥J\left(V\right)∥}_{2}}$ or Histogram Intersection $S_{\mathrm{hist}}\left(M\left(V\right),J\left(V\right)\right)=\frac{\sum_{i=1}^{n}\min\left(M{\left(V\right)}_{i},J{\left(V\right)}_{i}\right)}{\sum_{i=1}^{n}M{\left(V\right)}_{i}}$ evaluate proportional angular and magnitude alignment, offering smooth continuous measures robust to minor variations in event counts.

The similarity measure Sim(G) between the pattern feature vectors M(G) and J(G) is calculated using a ratio of minimum and maximum bin intensities across the γ temporal bins:

(7) $$\mathrm{Sim}\left(G\right)=\frac{\sum_{i=1}^{\gamma }\min\left(J{\left(G\right)}_{i},M{\left(G\right)}_{i}\right)}{\sum_{i=1}^{\gamma }\max\left(J{\left(G\right)}_{i},M{\left(G\right)}_{i}\right)+\epsilon }$$

where Sim(G)∈[0,1] represents the temporal correlation between the template and the live stream. A value of ≈1 signifies a perfect match in excitation patterns, while ϵ is a small constant included to prevent division by zero in instances of negligible event activity.

The global similarity score *U* is calculated by integrating the motion direction similarity Sim(V) and the temporal pattern similarity Sim(G) using a joint multiplicative formulation:

(8) U=Sim(G)·Sim(V)

In this formulation, U∈[0,1] provides a unified numerical metric representing the overall similarity between the candidate Motion of Interest *M* and the live stream segment *J*. By employing multiplicative feature fusion, a high global similarity score is achieved only when both directional motion flow (Sim(V)) and temporal excitation patterns (Sim(G)) exhibit strong alignment. If either component’s similarity is low (for instance, if Sim(G)=0.1 and Sim(V)=0.9), the global score is suppressed (U=0.09), correctly preventing false positive matches.

The classification decision for a live event stream LS is executed by sweeping a sliding temporal window *J* of length |J|=|M| with a stride of 1 ms. For multi-class motion recognition, the predicted class $\hat{k}$ is assigned using a nearest-neighbour maximum similarity rule:

(9) $$\hat{k}=\arg\max_{k}U\left(M_{k},J\right)$$

A detection threshold $\tau_{U}=0.65$ is applied to determine whether a valid gesture or motion event has occurred versus background activity. The overall operational parameters of the framework are summarised as follows:

- Spatio-Temporal Correlation Constraint: Temporal threshold Δ=5μs over a 3×3 Moore neighbourhood *H*.
- Motion Vector Bins: n=8 directional arc bins (45° resolution).
- Pattern Bin Optimisation: Temporal bin width β computed per class using Poisson cost minimisation (Equation (5)).
- Numerical Stability Offset: $\epsilon =10^{-7}$.
- SVM Classifier Configuration: For the SVM baseline experiments in Section 2.2 and Section 3, a Radial Basis Function (RBF) kernel SVM is trained with penalty parameter C=1.0, auto kernel scale $\gamma =1/n_{\mathrm{features}}$, and a One-vs-One multi-class strategy. Input feature vectors are normalised to [0,1] via min–max scaling. Models are trained on a 70:30 train–test split, with hyperparameters validated via 5-fold cross-validation grid search.

## 3. Results

The P-TED classification framework was evaluated across three distinct domains to demonstrate its robustness, speed, and accuracy compared to both state-of-the-art neuromorphic descriptors and modified frame-based techniques. Following the framework described in Section 2.2, three experiments were conducted to assess P-TED’s ability to recognise distinct motion profiles. The “Motions of Interest” (MoIs) remained constant across evaluations, with no variation in scale or orientation unless explicitly specified. For each class, the bin size β was set to the optimal value derived from the respective MoI statistics.

### 3.1. Object and Character Recognition Experiment

**Figure 4:**
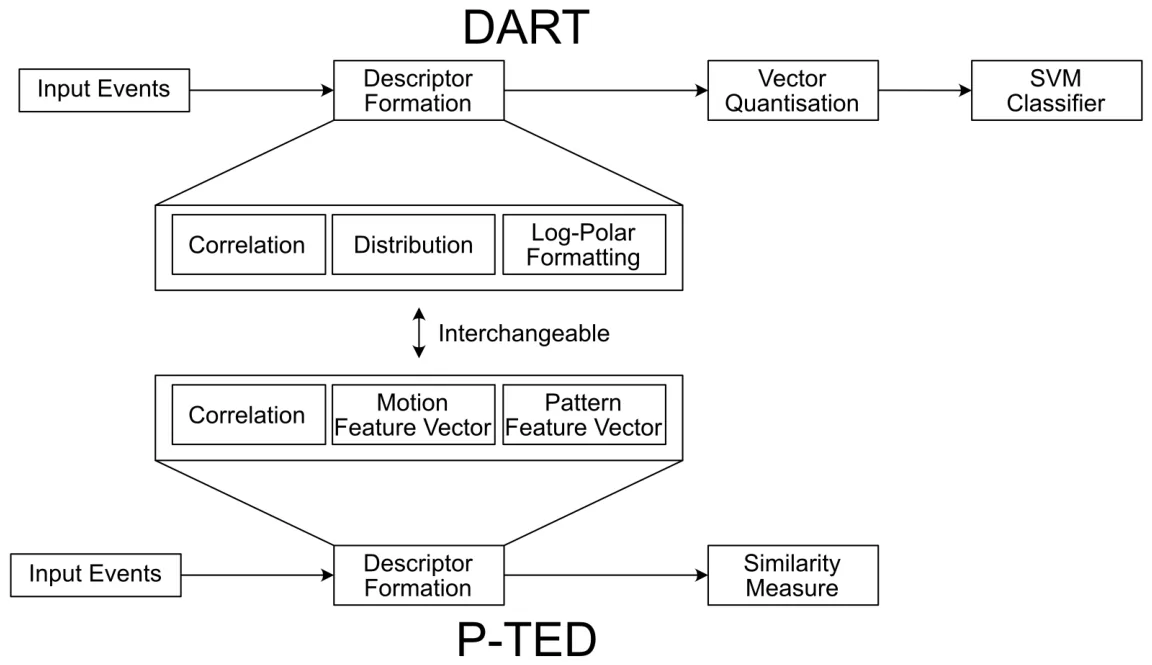
Architectural comparison between the DART and P-TED frameworks, highlighting the modular segments that allow for descriptor interchangeability.

The P-TED classification framework is utilised for both object and character recognition, and its performance was evaluated against the Distribution-Aware Retinal Transform (DART) framework [15]. DART is a state-of-the-art event-based descriptor designed to represent motion structural context by encoding event streams into log-polar grids. This approach simulates the spatial distribution of cone photoreceptors found within the primate fovea [36]. Unlike the threshold-based comparison used in P-TED, DART employs a machine learning pipeline, typically requiring a Support Vector Machine (SVM) to interpret the descriptors. While the original implementation in [15] did not focus on optimisation, the DART pipeline used in this study was enhanced via an exhaustive grid search for hyperparameter tuning. P-TED is compared against this established framework to demonstrate the efficacy of manual feature engineering over deep learning or complex learning pipelines, particularly in scenarios defined by low latency and stringent power constraints. Figure 4 illustrates the architectural differences and the interchangeable segments between the DART and P-TED frameworks.

**Figure 5:**
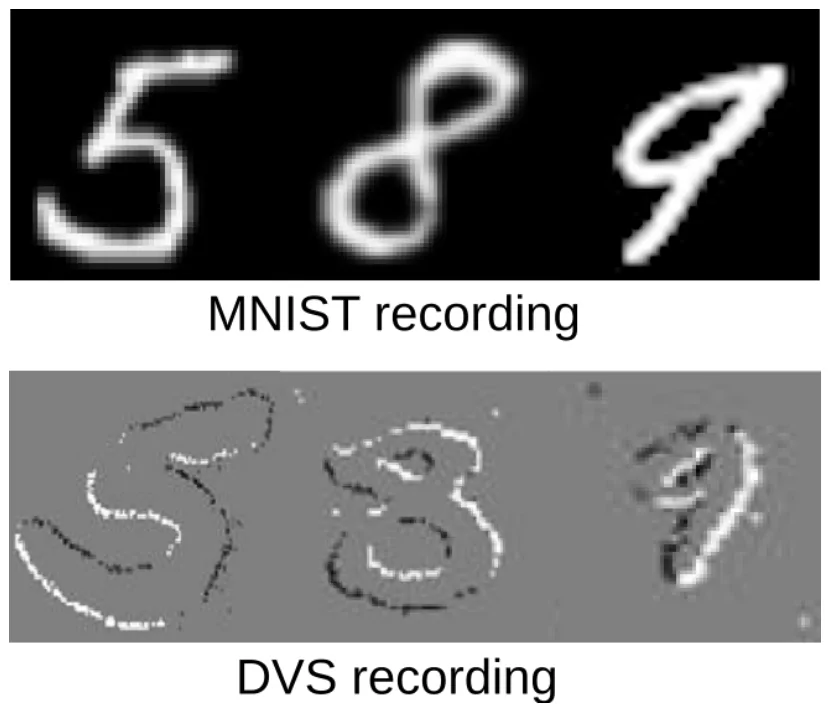
Visual samples of entries 5, 8, and 9 from the standard MNIST dataset alongside their corresponding MNIST-DVS event stream representations.

**Figure 6:**
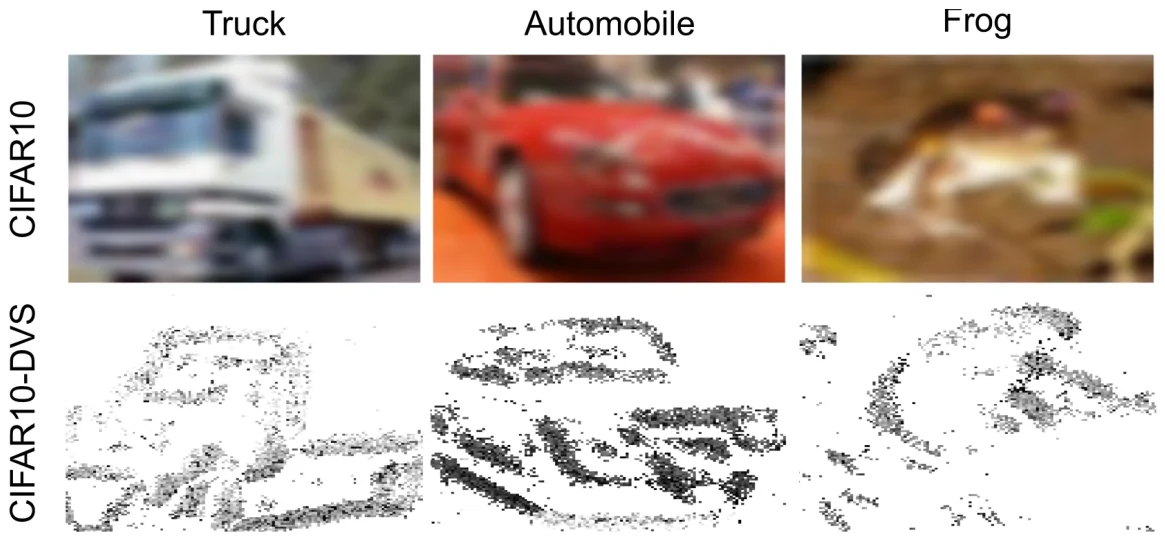
Samples of the truck, automobile, and frog classes from the CIFAR-10 dataset and their recorded CIFAR10-DVS counterparts.

In this experiment, P-TED and DART were compared using the MNIST-DVS [37] and CIFAR10-DVS [38] datasets. The MNIST-DVS dataset consists of 30,000 event streams obtained by recording moving handwritten digits on an LCD monitor, with each recording lasting approximately 2–3 s. Figure 5 illustrates samples of the digits 5, 8, and 9 in both their original and event-based formats. The CIFAR10-DVS dataset contains 10,000 event streams across 10 classes (e.g., airplanes, birds, frogs), recorded using a closed-loop smooth movement protocol. Samples for the truck, automobile, and frog classes are shown in Figure 6. The evaluation employed a cross-comparison methodology to isolate the performance of the descriptors from their respective matching frameworks (Figure 4). First, the P-TED replaced the DART descriptor within the SVM-based learning pipeline. Second, the DART descriptor was integrated into the P-TED threshold-based classification framework.

The results in Table 1 indicate that, while DART’s learning-heavy pipeline achieves higher peak accuracy on complex datasets, P-TED offers a significant 3.8 × speedup in classification latency. This efficiency confirms P-TED’s suitability for resource-constrained, real-time neuromorphic systems where temporal responsiveness is prioritised.

Table 2 presents the classification performance using the P-TED similarity metric (Equation (8)) for both descriptors. For these experiments, the first occurrence of each class served as the MoI template, while the remaining samples provided the testing set for accuracy calculation. The results demonstrate that the combination of the P-TED and the P-TED similarity framework yields the highest recognition performance. The P-TED framework is specifically architected to leverage the decoupled feature vectors (*V* and *G*); consequently, the DART descriptor, which does not capture temporal excitation patterns in the same manner, is less optimal for this framework. While the standard DART descriptor relies heavily on the spatial–structural relationships of events during the correlation and transform stages, P-TED limits spatial constraints to the initial correlation phase. By representing motion direction and firing patterns as separate entities rather than conjoining them, P-TED captures a more robust signature of the underlying asynchronous stimulus.

Conversely, Table 3 presents the results using the DART SVM-based pipeline. To establish a rigorous baseline, an optimised version of DART was included, utilising hyperparameter tuning via exhaustive grid search. All SVM configurations employed a 70:30 training-to-testing ratio. The results in Table 3 indicate that integrating the P-TED into the DART framework yields the highest recognition performance. This improved accuracy is likely attributable to the compact nature of the P-TED feature vectors, which allow the SVM learning pipeline to identify discriminative boundaries more effectively than a direct similarity measure. However, this performance gain involves a significant trade-off in computational efficiency. During experimental testing, the DART framework required 10.3 ms to classify samples from the MNIST-DVS and CIFAR10-DVS datasets (averaged over five runs). In contrast, the P-TED framework achieved classification in only 2.7 ms. This discrepancy highlights the substantial computational overhead inherent in machine learning-based matching, such as SVM inference, compared to the streamlined P-TED threshold-based framework.

To isolate the source of the speed advantage and address whether latency gains stem from descriptor design or classifier matching strategy, we decoupled the processing pipeline into feature extraction latency ($T_{\mathrm{feat}}$) and classifier matching latency ($T_{\mathrm{match}}$), as detailed in Table 4.

As shown in Table 4, P-TED feature extraction itself requires only 1.90 ms compared to 8.10 ms for DART, yielding a 4.26× speedup solely from descriptor formation. This advantage arises because P-TED avoids complex logarithmic foveal grid projections, relying instead on lightweight Moore neighbourhood lookups and 1D temporal rate binning. When combined with the threshold-based matching framework ($T_{\mathrm{match}}=0.80$ ms), the total latency is 2.70 ms, representing a 3.81× overall speedup compared to DART with the SVM (10.30 ms).

### 3.2. Mobile Robot Motion Experiment

**Figure 7:**
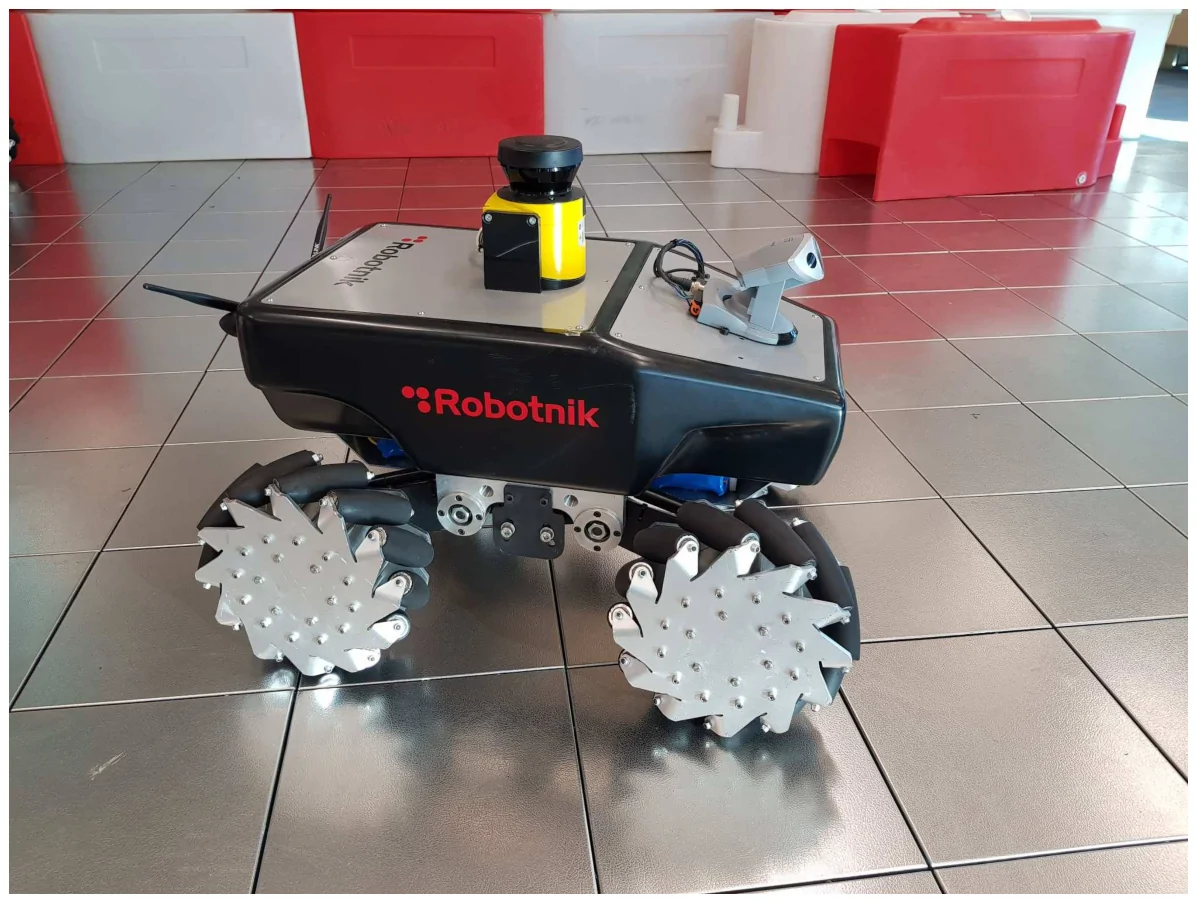
The Robotnik Summit XL mobile platform used in the robotic scaling and compounded motion experiments.

To evaluate P-TED in a real-world dynamic environment, we utilised a Robotnik Summit XL mobile platform (equipped with four-wheel skid-steer drive motors and internal wheel encoders) [39], as illustrated in Figure 7, observed by an off-board DAVIS240C event-based sensor mounted on a static tripod to record high-temporal-resolution visual motion streams. The robot was manually controlled to perform four cardinal movements, forward, backward, left, and right, which were captured by the static DAVIS240C sensor. P-TED was benchmarked against Modified Space-Time Template Matching (M-STTM), a frame-based system [40] adapted for event-based data by ingesting event frames generated through temporal event summation [41,42].

The kinematic manoeuvres of the Summit XL, defined by its linear velocity and angular velocity, map directly to the P-TED feature vectors. Pure translational kinematics generate directional trajectories captured by the spatial Moore neighbourhood arc counts in the motion feature vector (*V*), while velocity dynamics govern the rate of luminance changes, shaping the temporal firing density signatures encoded in the pattern feature vector (*G*).

**Figure 8:**
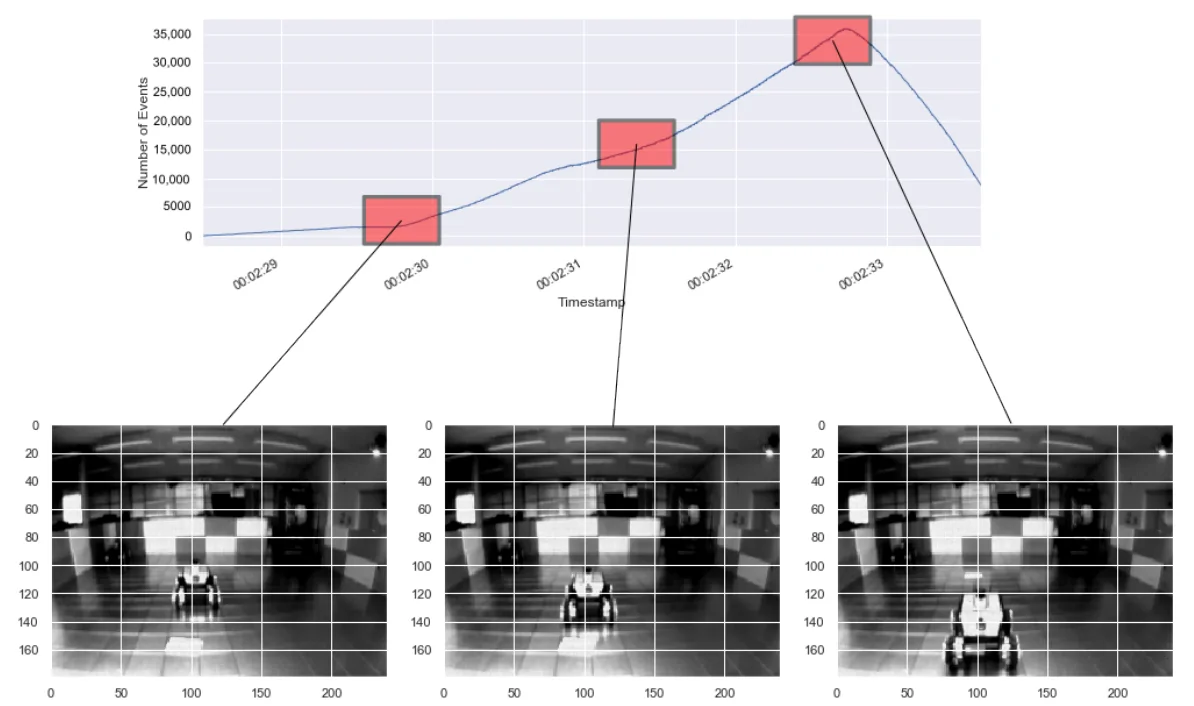
Illustration of the Summit XL performing a forward motion. The sequence demonstrates the increase in event response and pixel excitation as the robot’s scale increases relative to the static DAVIS240C sensor. The blue line represents the number of events over time.

The evaluation was conducted in two stages: isolated directional movements and a “compounded motion” stream (forward, right, then backward) using multiple MoI processes in parallel. This setup allowed for the assessment of two primary challenges in asynchronous motion recognition:

- Scale Invariance and Robustness: As the robot moves closer to the camera, the event response increases non-linearly due to the changing scale (see Figure 8). While the absolute number of events fluctuates significantly with proximity, P-TED maintains high similarity scores during forward and backward manoeuvres. This demonstrates a degree of scale invariance derived from the optimised temporal binning (β) and the rate encoding approach, which focuses on temporal dynamics rather than raw event volume.
- Symmetry and Mirroring: During the compounded motion phase, the “right” motion was deliberately omitted from the live stream to test for false positives. Structural frame-based descriptors like M-STTM frequently fail in this scenario, detecting a “Right” motion during a “Left” movement due to spatial mirroring. In contrast, P-TED’s motion vector *V* successfully differentiates these symmetric movements, yielding only a negligible similarity increase for non-occurring directions.

**Figure 9:**
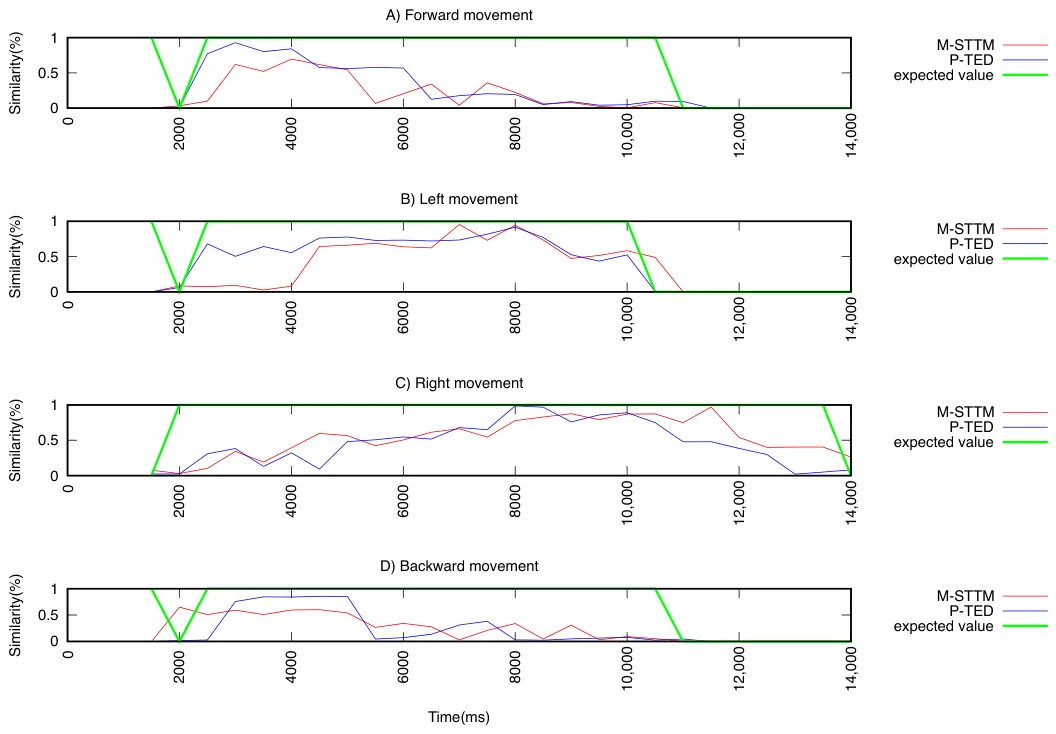
Similarity results from four individual robotic movement tests (forward, left, right, backward).

Figure 9 illustrates the similarity values obtained during one of the five trials for the one-versus-one individual motion experiment. These results are summarised in Table 5, which provides the Area Under the Curve (AUC) values for both methods. For the results presented in Table 5, an ideal performance is represented by a value of 1.0. The comparative analysis indicates that P-TED consistently performs favourably against M-STTM in all but a single case. This robust performance across isolated manoeuvres suggests that the decoupled motion and pattern vectors remain more stable than frame-based templates when subjected to the inherent noise and asynchronous nature of robotic event data.

**Figure 10:**
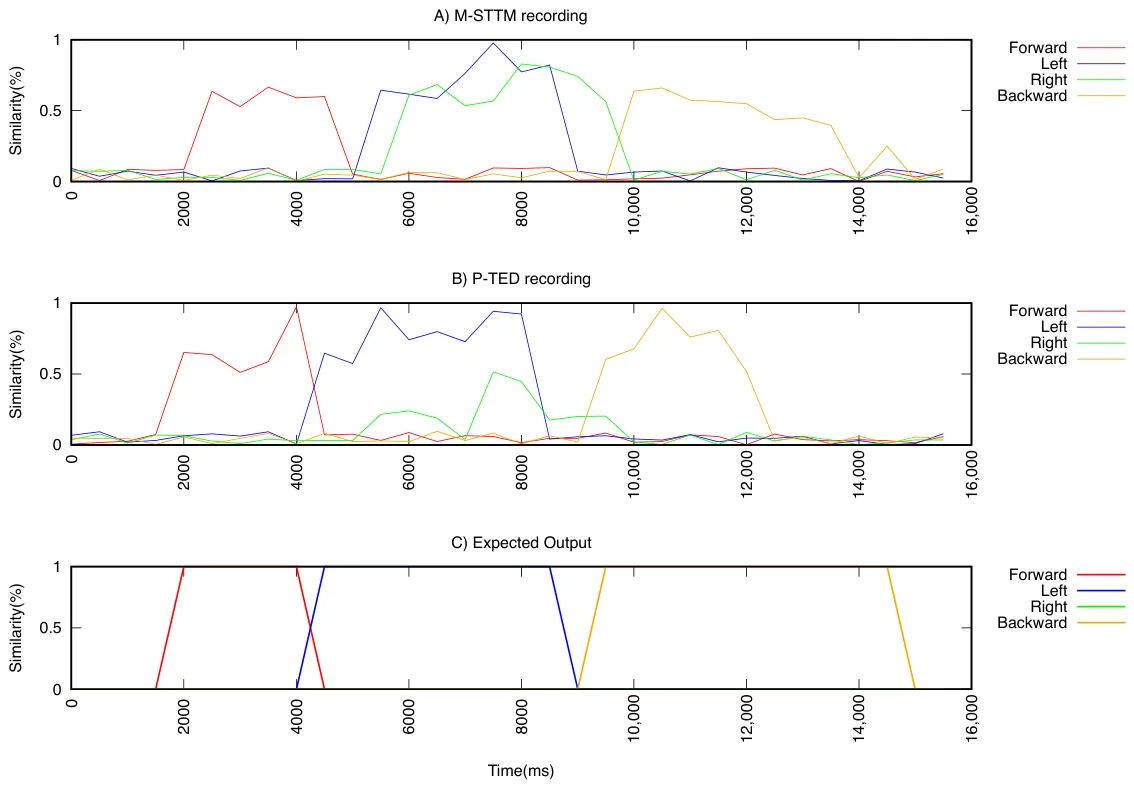
Similarity results from compound robot movement.

Figure 10 illustrates the similarity values recorded during the compounded motion experiment. These results are summarised in Table 6 using AUC metrics derived from the plot. For all entries in Table 6, an ideal classification score is 1.0. The comparative results demonstrate that P-TED achieves performance comparable to M-STTM, effectively validating its capability to recognise complex, multi-stage motions without the computational overhead of converting event data into intermediate frames. Notably, P-TED shows superior performance in the forward and left manoeuvres, while M-STTM retains a marginal advantage in backward motion.

Figure 10 illustrates the similarity trajectories for the M-STTM and P-TED systems, respectively. These results demonstrate that P-TED achieves a level of performance comparable to M-STTM while operating directly on asynchronous event data, thereby eliminating the computational latency associated with frame-based transformations. A critical distinction observed in the results is M-STTM’s susceptibility to false detections caused by spatial mirroring. As evidenced in Figure 10, the M-STTM framework erroneously indicates significant similarity for the “Right” motion (green line) during a “Left” movement. In contrast, P-TED exhibits superior directional disambiguation; the similarity scores for the non-occurring “Right” motion show only a negligible increase. This confirms that P-TED’s motion feature vector *V* is more robust than structural intensity-based templates when differentiating between symmetric motion profiles.

### 3.3. Robotic Hand RoShamBo Experiment

**Figure 11:**
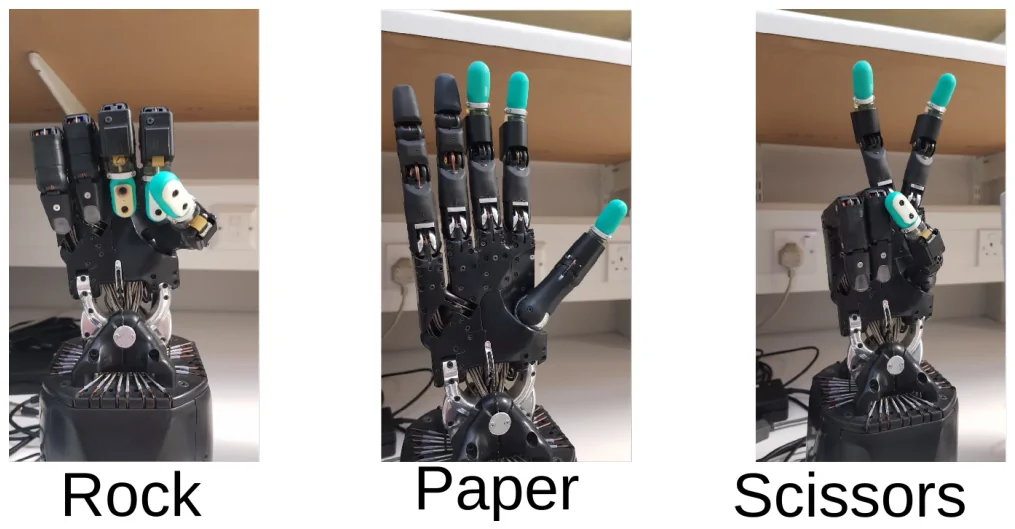
The three RoShamBo position states (Rock, Paper, and Scissors) performed by the Shadow Robot Dexterous Hand, annotated with average model recognition probabilities (Rock: 0.88, Paper: 0.96, Scissors: 0.94).

The third experiment utilised the Shadow Robot Company Dexterous Hand [43,44] to evaluate performance in complex, non-rigid motion scenarios. This study was inspired by prior research [5] which employed a CNN-based solution to classify hand symbols for the game “RoShamBo” (Rock, Paper, Scissors), as illustrated in Figure 11. Firstly, the Shadow Hand performed three distinctive motions, Rock, Paper, and Scissors, which were captured to construct the corresponding MoI templates for each symbol. Following template construction, the hand performed the symbols in a cyclic sequence (Rock, Paper, Scissors, repeated) for a total of five occurrences per symbol. The P-TED MoIs were integrated with the SVM classification framework (Section 2.2) to identify the live gestures and determine the correct competitive response based on game rules. For instance, upon recognising a “Paper” gesture, the framework could accurately generate a “Scissors” response. This experiment serves to demonstrate the framework’s robustness in recognising specific motion signatures within a continuous, high-entropy event stream containing both symbol formation and inter-gesture transitions.

The results of this experiment, presented in Table 7, detail the total occurrences for each RoShamBo symbol alongside the corresponding counts for successful detections and misses. A valid detection was defined as a majority output, with the final output remaining correct immediately prior to the gesture transition within a 5 ms temporal window before the Shadow Hand initiated the subsequent motion. The gesture recognition confidence scores (probabilities) obtained from the model were recorded as P(Paper)=0.96, P(Rock)=0.88, and P(Scissors)=0.94, demonstrating high classification certainty. The results in Table 7 demonstrate that the P-TED classification framework is highly capable of recognising distinct robotic hand gestures. Notably, this recognition was achieved over a continuous live event stream that included both the stable formation of the symbols and the high-entropy events generated during inter-symbol transitions. The successful classification during these transitions illustrates the robustness of the framework in differentiating meaningful motion signatures from the transient noise inherent in complex, multi-joint robotic movements.

### 3.4. Ablation Study and Feature Necessity Analysis

To empirically isolate the individual contributions of the motion feature vector (*V*) and the pattern feature vector (*G*), an ablation study was conducted across the three experimental domains. Table 8 compares classification accuracy obtained when relying exclusively on the motion direction (*V*-only), excitation pattern (*G*-only), and combined dual-vector representation (U=Sim(G)·Sim(V)).

The empirical findings in Table 8 confirm the necessity of the dual-vector formulation:

- V-only: Evaluates directional flow across the Moore neighbourhood. While effective for simple translational motions, it lacks temporal firing density signatures, leading to reduced accuracy on complex gestures and characters.
- G-only: Captures temporal rate excitation profiles. While it provides strong class discriminability based on firing dynamics, it lacks spatial–directional specificity, rendering it vulnerable to directional ambiguity.
- Combined Dual-Vector (U): Fuses spatial–directional flow and temporal rate dynamics. The synergy between V and G yields substantial performance improvements of +12.48% on MNIST-DVS and +13.33% on RoShamBo over the single best individual feature vector, validating the dual-vector architecture.

## 4. Discussion

This paper introduced the Post-Stimulus Time-Dependent Event Descriptor (P-TED), a “pure event” framework designed for the asynchronous processing of neuromorphic vision data. By bypassing traditional event-to-frame transformations, P-TED maintains the low-latency and high-temporal-resolution advantages inherent to event-based sensors. Our evaluation across three distinct experimental domains yielded several key findings regarding the framework’s efficacy:

- Computational Efficiency and Decoupled Speed Advantage: Comparison with the state-of-the-art DART framework on the MNIST-DVS and CIFAR10-DVS datasets revealed that P-TED achieved a classification latency of 2.7 ms. Decoupled latency profiling confirmed that P-TED feature extraction required only 1.90 ms (4.26× faster than DART’s 8.10 ms), proving that the speed advantage stems fundamentally from the lightweight descriptor design rather than merely the threshold matching strategy.
- Directional Robustness and Symmetry: In mobile robotics experiments using the Summit XL platform, P-TED successfully distinguished between symmetric and mirrored movements. While the frame-based M-STTM baseline suffered from false positives during mirrored manoeuvres, P-TED’s motion feature vector *V* provided robust directional disambiguation.
- Scale Invariance: The framework demonstrated partial scale invariance, effectively recognising motion signatures even as the robot’s proximity to the sensor caused non-linear fluctuations in event density.
- Complex Gesture Recognition: Evaluation with the Shadow Robot Dexterous Hand demonstrated P-TED’s ability to classify non-rigid gestures (RoShamBo) from a live event stream. The system maintained high reliability even during the high-entropy transition phases between symbol formations.

### Methodological Considerations, Limitations, and Future Directions

While P-TED exhibits strong low-latency performance, several technical aspects and limitations warrant deeper discussion:

1. Dynamic Event Rate Sensitivity and Stationary Assumptions: The optimal bin size β calculation (Equation (5)) relies on Poisson statistics under an assumption of stationary event arrival rates over the Motion of Interest (MoI) window. In scenarios involving highly non-stationary dynamics (e.g., erratic camera acceleration or sudden speed changes), fixed binning can lead to sub-optimal quantisation. Implementing an online adaptive temporal binning scheme, where β(t) dynamically adjusts according to instantaneous event arrival rate variance R(t), represents an important direction for future research.

2. Generalisability to Unstructured Real-World Environments: Benchmark datasets (MNIST-DVS, CIFAR10-DVS) and laboratory motion sequences serve to isolate descriptor mechanics, but unconstrained real-world deployment introduces challenges such as dynamic background clutter, moving distractor objects, occlusions, and ambient illumination shifts. Although neuromorphic event sensors possess a high dynamic range (>120 dB) that mitigates global lighting variations, background clutter generates spurious event spikes. Extending P-TED with spatio-temporal Background Activity Filters (BAF) and motion-based Region of Interest (ROI) tracking will be critical for unconstrained robotic deployment.

3. Handcrafted Dual-Vector Features vs. Learned Representations: A central debate in event-based computer vision concerns handcrafted features versus deep learning representations (e.g., Spiking Neural Networks like SLAYER or lightweight Convolutional Neural Networks on event frames). While learned representations achieve superior peak accuracy on complex, high-entropy benchmarks (e.g., CIFAR10-DVS), they incur substantial trade-offs: large memory footprints (>10 MB), requirement for offline training, non-deterministic explainability, and reliance on GPU/NPU hardware accelerators [13,21]. In contrast, P-TED’s handcrafted dual-vector formulation requires zero offline training data, operates with an ultra-compact memory footprint (<5 KB), executes with microsecond latency (2.70 ms) on low-power embedded microcontrollers without specialised hardware, and provides fully explainable directional and temporal components.

4. Continuous Distance Metrics: While the discrete matching metric Sim(V) in Equation (6) provides integer-level efficiency for hardware implementation, continuous metrics such as Cosine Similarity and Histogram Intersection offer smoother similarity manifolds and higher resilience to minor counting noise, providing a flexible trade-off for software-based robotic vision frameworks.

## 5. Conclusions

This paper presented the Post-Stimulus Time-Dependent Event Descriptor (P-TED), a novel pure event feature extraction and classification framework for asynchronous neuromorphic vision sensors. By operating directly on microsecond event streams through a spatio-temporal Moore neighbourhood correlation and rate-encoded temporal binning, P-TED avoids the computational latency and temporal information loss inherent in frame-based conversions.

Extensive benchmarking across object recognition (MNIST-DVS, CIFAR10-DVS), mobile platform movement (Summit XL), and non-rigid robotic hand gestures (RoShamBo) demonstrated the following core outcomes:

1. Low Latency: P-TED achieved a processing latency of 2.70 ms (3.81× faster than DART with SVM), driven by a 4.26× speedup in feature extraction (1.90 ms vs 8.10 ms).
2. Directional Disambiguation: The motion direction vector *V* resolves spatial mirroring ambiguity, eliminating false positive detections observed in frame-based space–time templates.
3. Empirical Dual-Vector Synergy: Ablation studies confirmed that combining motion direction (*V*) and pattern excitation (*G*) via joint multiplicative fusion (U=Sim(G)·Sim(V)) improves recognition accuracy by +12.48% over single-vector representations.

Overall, P-TED provides an efficient, explainable, and ultra-low-latency foundation for real-time neuromorphic perception in resource-constrained robotic systems. It should be noted that robustness to non-stationary event rates, clutter, occlusion, and illumination changes has not been experimentally validated.

Future work will extend the framework with online adaptive binning, background activity filtering, and ego-motion compensation for unstructured outdoor operations.

## Figures and Tables

**Table 1:** Comparative accuracy and latency on MNIST-DVS and CIFAR10-DVS.

Dataset	Descriptor	Framework	Acc. (%)	Lat. (ms)
MNIST-DVS	P-TED	P-TED	91.73	2.70
	DART	DART (Opt)	98.11	10.30
CIFAR10-DVS	P-TED	P-TED	58.55	2.70
	DART	DART (Opt)	65.93	10.30

**Table 2:** Accuracy results from using the P-TED framework.

Dataset	P-TED (%)	DART (%)	Time (ms)
MNIST-DVS	91.73	86.74	2.70
CIFAR10-DVS	58.55	51.74	2.70

**Table 3:** Accuracy results from using the DART framework (%).

Dataset	P-TED	DART	DART Opt.	Time (ms)
MNIST-DVS	98.31	97.95	98.11	10.30
CIFAR10-DVS	66.90	65.78	65.93	10.30

**Table 4:** Decoupled processing latency breakdown (ms): feature extraction vs. classifier matching.

Descriptor	Matching Framework	$T_{\mathrm{feat}}$ (ms)	$T_{\mathrm{match}}$ (ms)	Total Latency (ms)
P-TED	P-TED (Threshold)	1.90	0.80	2.70
P-TED	DART (SVM)	1.90	2.20	4.10
DART	P-TED (Threshold)	8.10	0.80	8.90
DART	DART (SVM)	8.10	2.20	10.30

**Table 5:** Individual motion results showing the AUC of the responses shown in Figure 9 for M-STTM and P-TED.

Motion	M-STTM (AUC)	P-TED (AUC)
Forward	0.288	0.402
Left	0.546	0.777
Right	0.574	0.496
Backward	0.331	0.349

**Table 6:** Compound motion results: AUC comparison of M-STTM and P-TED responses corresponding to Figure 10.

Motion	M-STTM (AUC)	P-TED (AUC)
Forward	0.560	0.725
Left	0.630	0.855
Right	0.611	0.209
Backward	0.453	0.376

**Table 7:** Shadow Hand RoShamBo experiment results: comparison of symbol occurrences, successful detections, and misses.

RoShamBo Symbol	Occurrences	Detected	Missed
Paper	5	5	0
Rock	5	4	1
Scissors	5	5	0

**Table 8:** Ablation study: recognition accuracy (%) for individual features vs. dual-vector P-TED (*U*).

Dataset/Task	Motion Vector *V* Only (%)	Pattern Vector *G* Only (%)	Dual-Vector P-TED *U* (%)
MNIST-DVS	71.40	79.25	91.73
CIFAR10-DVS	42.10	48.60	58.55
RoShamBo Gestures	66.67	80.00	93.33

## Data Availability

The raw data supporting the conclusions of this article will be made available by the authors on request.
